# Appraising patient preference methods for decision-making in the medical product lifecycle: an empirical comparison

**DOI:** 10.1186/s12911-020-01142-w

**Published:** 2020-06-19

**Authors:** Chiara Whichello, Bennett Levitan, Juhaeri Juhaeri, Vaishali Patadia, Rachael DiSantostefano, Cathy Anne Pinto, Esther W. de Bekker-Grob

**Affiliations:** 1 Erasmus School of Health Policy & Management and Erasmus Choice Modelling Centre, Erasmus University Rotterdam, P.O. Box 1738, 3000 DR Rotterdam, The Netherlands; 2 Janssen R&D, LLC, Titusville, NJ USA; 3 Sanofi, Bridgewater, NJ USA; 4 Merck & Co, Inc, Kenilworth, NJ USA

**Keywords:** Patient preferences, Preference elicitation, Preference exploration, Preference assessment, Method comparison, Decision-making, Medical product lifecycle, Health preference research, Patient preference study

## Abstract

**Background:**

Incorporating patient preference (PP) information into decision-making has become increasingly important to many stakeholders. However, there is little guidance on which patient preference assessment methods, including preference exploration (qualitative) and elicitation (quantitative) methods, are most suitable for decision-making at different stages in the medical product lifecycle (MPLC). This study aimed to use an empirical approach to assess which attributes of PP assessment methods are most important, and to identify which methods are most suitable, for decision-makers’ needs during different stages in the MPLC.

**Methods:**

A four-step cumulative approach was taken: 1) Identify important criteria to appraise methods through a Q-methodology exercise, 2) Determine numerical weights to ascertain the relative importance of each criterion through an analytical hierarchy process, 3) Assess the performance of 33 PP methods by applying these weights, consulting international health preference research experts and review of literature, and 4) Compare and rank the methods within taxonomy groups reflecting their similar techniques to identify the most promising methods.

**Results:**

The Q-methodology exercise was completed by 54 stakeholders with PP study experience, and the analytical hierarchy process was completed by 85 stakeholders with PP study experience. Additionally, 17 health preference research experts were consulted to assess the performance of the PP methods. Thirteen promising preference exploration and elicitation methods were identified as likely to meet decision-makers’ needs. Additionally, eight other methods that decision-makers might consider were identified, although they appeared appropriate only for some stages of the MPLC.

**Conclusions:**

This transparent, weighted approach to the comparison of methods supports decision-makers and researchers in selecting PP methods most appropriate for a given application.

## Background

The integration of patient preferences into decision-making is becoming progressively more important throughout the medical product life cycle (MPLC) [1]. Patient preference (PP) information is defined by the United States (US) Food and Drug Administration (FDA) as: “qualitative or quantitative assessments of the relative desirability or acceptability to patients of specified alternatives or choices among outcomes or other attributes that differ among alternative health interventions” [2]. The Center for Devices and Radiological Health (CDRH) at the FDA has published guidance [3–5] on conducting preference studies and is soliciting research priorities in patient preference studies [6]. The European Medicines Agency (EMA) has published similar guidance and intention to investigate PP methodologies [7]. Other projects such as the MDIC (Medical Device Innovations Consortium [8] are promoting the importance of PP information in benefit-risk assessments, while the National Institute for Health and Care Excellence (NICE) is establishing patient preference research partnerships [9].

Overall, there is a consensus among stakeholders, including industry, health technology assessment (HTA) bodies or payers, regulatory authorities, and clinicians, that the use of PP information in decision-making for medical products might be beneficial to inform benefit-risk and HTA/payer assessments [10]. Despite this consensus, the results of PP studies are currently not integrated into the MPLC systematically. Generally, there is a lack of guidance in current literature regarding the choice of PP study methods [11]. As the role of PP information in decision-making increases, it is vital that decision-makers are able to select the most appropriate methods suitable for their requirements.

A total of 33 methods have been identified in contemporary literature [12] as being able to measure patient preferences in medical treatments. This includes 10 preference ‘exploration’ methods that collect descriptive data through the subjective experiences and decisions of participants (generally qualitative techniques), and 23 preferences ‘elicitation’ methods that collect quantifiable data able to be reported through statistical inferences or analysis (generally quantitative techniques) (Table 1). An appraisal of these numerous and diverse PP methods will aid in combatting the uncertainty that stakeholders face regarding which methods to use.

**Table 1:** Thirty-three patient preference exploration and elicitation methods (adapted from Soekhai et al. [12])

		**Methods**
Preference exploration methods	Individual methods	In-depth individual interviews
		(Semi) structured - individual interviews
		Complaints procedures
		Concept mapping^a^
	Group methods	Delphi method
		Dyadic interview
		Citizens’ juries
		Focus groups
		Nominal group technique
		Public meetings
Preference elicitation methods	Discrete choice based methods	Adaptive conjoint analysis
		Discrete choice experiment / Best- worst scaling (type 3)
	Indifference methods	Contingent valuation
		Person-trade off
		(Probabilistic) threshold technique
		Standard gamble
		Starting known efficacy
		Test trade-off
		Time trade-off
	Rating methods	Allocation of points
		Analytic hierarchy process
		Constant sum scaling
		Measure of value
		Outcome prioritization tool
		Repertory grid method
		Swing weighting
		Visual analogue scale
	Ranking methods	Best-worst scaling (type 1)^b^
		Best- worst scaling (type 2)^b^
		Control preference scale
		Q-methodology
		Qualitative discriminant process
		Self-explicated conjoint

^a^Concept mapping can be utilised as a group method, but for the purpose of this method comparison it will be taxonomised as an individual method because the success of its data collection is not dependent on the present of multiple participants, unlike the other group techniques

^b^Soekhai et al. [12] condensed Best-worst scaling types 1 and 2 into one method for the systematic review, but these were separated for this investigation to determine whether they performed differently

There is currently no comprehensive comparison between these methods, nor any generally assessment of which are most suitable for particular stages in the MPLC or for particular study design considerations. This study proposes a means of choosing between methods eliciting and exploring patient preferences. It aims to 1) identify criteria most important for appraising preference PP methods, with relative weights for those criteria and 2) identify PP methods most suitable for satisfying decision-makers’ needs in the MPLC.

## Methods

**Fig. 1:**
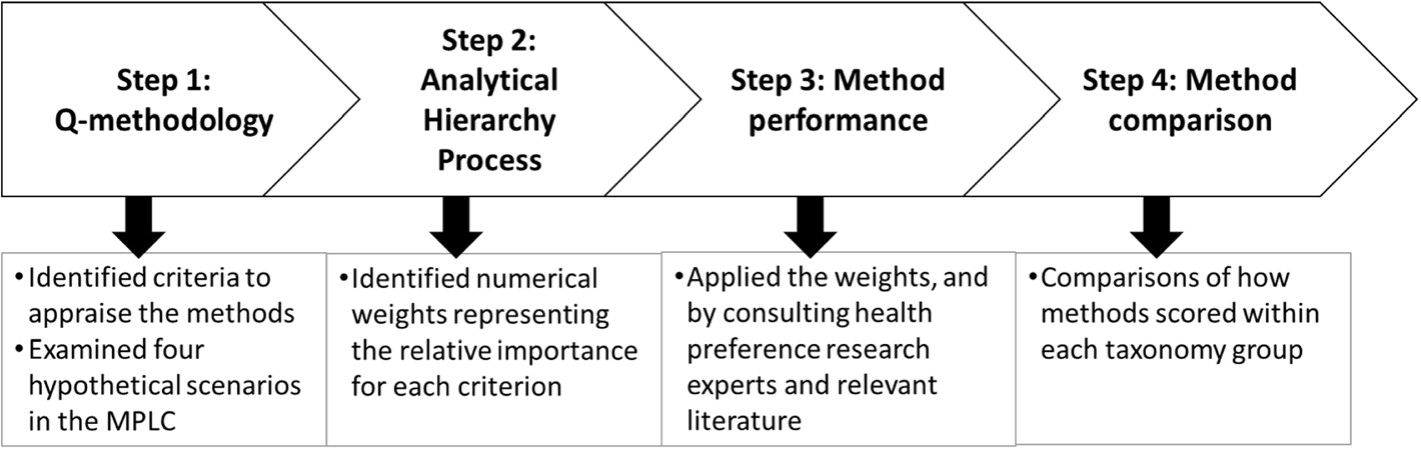
Study method process

In this study, a four step approach was taken (Fig. 1): 1) Identify important criteria to appraise methods through a Q-methodology exercise, 2) Determine numerical weights to ascertain the relative importance of each criterion through an analytical hierarchy process (AHP), 3) Assess the performance of 33 PP methods by applying these weights, consulting international health preference research (HPR) experts and by review of literature, and 4) Compare and rank the methods within taxonomy groups [12] reflecting their generally similar techniques to identify the most promising methods. This will result in the means of identifying the preference elicitation and exploration methods most suitable for decision-makers needs.

### Step 1: Q-methodology

To determine which criteria were most important when selecting a preference exploration or elicitation method in the MPLC, 35 initial criteria were developed based on previous studies, including MDIC’s patient-centred benefit-risk framework [8] and a systematic review by Ryan et al. [13]. These criteria can be found in Table 2. Q-methodology, a research technique using a unique ranking system [14–16] was used to rank these criteria. Q-methodology aims to capture the subjectivity of participants’ opinions or preferences by identifying correlations between participants across a sample of variables (in this case, the criteria), allowing researchers to contrast different attitudes of participants. It is an effective method even with samples under 100 participants. Q-methodology was used to identify similar viewpoints across diverse stakeholder groups, and to identify a ‘shortlist’ of the most important attributes from the participants’ rankings.

**Table 2:** Q-methodology results

**Most important criteria**	A: Early development	B: Early development	C: Late phase III	D: Post-marketing
A typical survey can be conducted at relatively low costs	✓	✓		
Data can be collected during quick sessions with participants		✓	✓	
Low frequency of sessions required by patients	✓	✓	✓	
Relatively quick delivery of preparation, data collection, and analysis	✓	✓	✓	✓
A large number of attributes can be explored				✓
Suitable to study preferences in a small sample size	✓	✓		✓
A low cognitive load on patients	✓	✓	✓	✓
Does not need an education tool or preparatory instructions in order to enhance participant comprehension		✓	✓	
Publically acknowledged by your organisation’s guidelines as an acceptable method to study preferences			✓	✓
New attributes can be added without making prior results invalid	✓	✓	✓	
Can be used to collect data from more than one participant in a single session			✓	
The analysis can calculate risk attitudes, like risk tolerance, and calculate how value functions bend due to the presence of uncertainty in the participant	✓	✓	✓	✓
Explores the reasons behind a preference in detail	✓	✓	✓	✓
Can estimate weights for attributes	✓	✓	✓	✓
Estimates trade-offs that patients are willing to make among attributes	✓	✓	✓	✓
Can quantify heterogeneity in preferences	✓	✓	✓	✓
Internal validity can be established	✓	✓	✓	✓
External validity can be established	✓	✓	✓	✓
Outcomes can refer to a course of health over time (as opposed to a constant health state)			✘	✘
Sensitivity analysis is possible	✘	✘	✘	✘
Can combine quantitative and qualitative methods		✘	✘	✘
Applies validation tests	✘		✘	✘
Results can be reproduced by an (independent) researcher for reproducibility	✘	✘	✘	✘
Applies tests for consistency			✘	✘
Can be conducted without the need for specialized software (beyond Excel)				
Can be conducted without programming skills				
Researcher does not need to supervise the data collection				
Does not require hypothetical scenarios				
Attributes and attribute levels can be determined as part of the method itself (internal identification)				
Data saturation can be achieved relatively quickly				
Does not require model estimations				
Outcomes can be expressed in a particular format (e.g. probability scores, marginal rates of substitution, monetary values)				
Outcomes can refer to a constant health state (as opposed to a course of health over time)				
Uses respondent validation by asking participants to check their data or responses				
Validates through triangulation				

✓ Criteria considered important in the Q-methodology, included in the AHP

✘ Criteria considered important in the Q-methodology, but not included in the AHP for the following reasons: 1. The criterion does not sufficiently discriminate between each method (i.e. every method would perform the same way under the criterion), 2. The criterion reflects an element of good study conduct, and not a unique aspect of a method itself, 3. The criterion could be absorbed into other similar criteria, in order to avoid the oversaturation of themes

In a convenience sample, our participants consisted of academics, consultants conducting patient preference research for other stakeholders, HTA/payers, industry members, physicians, and patient organisation members, all with PP study experience. Participants were recruited from organisations partnered with the PREFER project [10], and also outside the project, through snowballing techniques, based on their experience with PP studies. In implementing Q-methodology, participants were first asked to assign our 35 criteria into three groups (most important, moderately important, least important), and then place these criteria on a V-shaped grid [14], visually ranking the criteria from most important (on the far right of the V-shape) to least important (on the far left). Participants completed this task for four hypothetical scenarios representing different stages of the MPLC, in which patient preferences could be required in order to inform a decision including: early development scenarios for products with mechanisms that are understood (Scenario A) and not understood (Scenario B), a late phase III scenario (Scenario C), and a post-marketing scenario (Scenario D) (see Table 3).

**Table 3:** MPLC Scenarios for Q-methodology and AHP

**MPLC Scenario**	**COLUMN A** **Q-Methodology Description**	**COLUMN B** **AHP Description**
A: Early Development (mechanism of action well understood)	Phase 2a results are complete and phase 2b is being designed. The indication and population are well-defined. The clinical and commercial teams are discussing the criteria and requirements for a target product profile (TPP), including which benefits, risks and tolerability issues to include and what levels of each are the target. The TPP decision is an in-house activity for now, with information being shared with commercial and clinical development teams**. The mechanism of action is well-understood. This is a novel indication of a treatment** that has been on the market for years.	A drug is being developed for a certain population. **The mechanism of action,** meaning the specific biochemical interaction by which a drug produces an effect, **is well-understood.** The drug has **been on the market for years** for a different condition and its benefit-risk profile is well-understood in that population. However, this is a **novel indication** of the treatment, and the **benefits, risks, and dosing strategy are still being identified** in the new population and condition. Phase 2a studies have been conducted to demonstrate clinical efficacy. Phase 2b studies are being designed to find the optimum dose that has the greatest efficacy with minimal side-effects. The internal clinical and commercial teams are discussing the criteria and requirements for a successful treatment. **The preference study would be conducted for internal decision-making on whether or not the medication should advance further in development.**
B: Early Development (mechanism of action is not well understood)	Phase 2a results are complete and phase 2b is being designed. The indication and population are well-defined. The clinical and commercial teams are discussing the criteria and requirements for a target product profile (TPP), including which benefits, risks and tolerability issues to include and what levels of each are the target. The TPP decision is an in-house activity for now, with information being shared with commercial and clinical development teams. **The mechanism of action is not understood. This is novel indication.**	A drug is being developed for a certain population. **The mechanism of action**, meaning the specific biochemical interaction by which a drug produces an effect, **is not understood**. This is **a novel indication of the treatment**, and **the benefits, risks, and dosing strategy are still being identified**. Phase 2a studies have been conducted to demonstrate clinical efficacy. Phase 2b studies are being designed to find the optimum dose that has the greatest efficacy with minimal side-effects. The clinical and commercial teams are discussing the criteria and requirements for a target product profile (TPP), including which benefits, risks and tolerability issues to include and what levels of each are the target. The TPP decision is an in-house activity for now, with information being shared with commercial and clinical development teams. **The preference study would be conducted for internal decision-making on whether or not the medication should advance further in development.**
C: Late Phase III	Clinical data available for pivotal trials. Mechanism of action is understood. Advisory committee/scientific advisory group meeting is scheduled. The goal is to provide data to support benefit-risk assessment to health authorities for regulatory dossier submission.	The **benefits and risks dosing strategy** of a medical product are reasonably **well-characterized**, as **clinical trials** in patients have been completed to assess efficacy, effectiveness, and safety. **Mechanism of action is understood**, (meaning the specific biochemical interaction by which a drug produces an effect). There is an advisory committee/scientific advisory group meeting scheduled. **The goal is to provide patient preference data to support benefit-risk assessment when submitting dossiers to regulators and HTA bodies.**
D: Post-Marketing	The treatment approved a year ago is now discovered from a registry or observational data to have a clinical significant side effect. Currently, the discussion is all in-house, but the signal is likely to lead to a discussion with health authorities.	A medical product **approved a year ago** is now discovered from a registry or observational data to have **a clinical significant side effect**. Currently, the discussion is all in-house, but the signal is likely to lead to a discussion with health authorities. **The preference study would be used to complement the clinical data by providing the patient’s perspective on benefit-risks.**

Participants were invited to participate in the online survey using the online application FlashQ [17] and randomly allocated two out of the four hypothetical scenarios. The data were analysed in the qmethod package [18] under R 3.4.1 software [19].

### Step 2: Analytical Hierarchy Process

To determine the relative importance of the criteria identified during Step 1, four Analytic Hierarchy Process (AHP) exercises were executed using Expert Choice 5.70 software [20]. AHP is a preference elicitation method which assesses the relative importance of attributes, with respect to achieving a goal, through pairwise comparisons. A block design [21] was used to reduce the number of pairwise comparisons, and therefore, the burden on each respondent. In each comparison, a participant indicates preference between two criteria and the strength of that preference on a 7-point scale [22, 23]. Based on these evaluations, a numerical weight can be derived for each attribute that reflects the relative importance of the criteria.

The same four hypothetical MPLC Scenarios A-D were used in the AHP as the Q-methodology, although the text was expanded for clarity, since the Q-methodology software demanded short texts on screen (Table 3). Several criteria that scored positively in the Q-methodology were not included in the AHP if the criterion: 1) did not sufficiently discriminate between each method (e.g. “results can be reproduced”), 2) reflected an element of good study conduct, and not a unique aspect of a method itself (e.g. “applies consistency tests”), 3) could be absorbed into other similar criteria, in order to avoid the oversaturation of themes (e.g. “applies validation tests”, which were absorbed into the criteria involving internal and external validity). This successfully improved the list of criteria for clarity and brevity, decreasing the cognitive burden on participants since the AHP required many pairwise comparisons. Additionally, the attribute of collecting data from more than one participant in a single session was expanded into two attributes, one regarding group dynamics and one regarding solitary exercises, because many methods are able to do both and we needed to determine if one setting was more important than the other in some circumstances.

A convenience sample of key stakeholders involved with PP studies including academics, consultants, HTA/payers, industry members, physicians, patient organisation members, and regulators, were invited to complete the AHP exercises online, and were randomly allocated two out of four hypothetical scenarios, including one of the early development scenarios, and one of the later scenarios (late phase III or post-marketing). Participants were recruited from organisations partnered with the PREFER project [10], and also outside the project, through snowballing techniques, based on their experience with PP studies.

### Step 3: Method Performance

This step assessed the performance of each of the 33 preference methods identified by Soekhai et al. [4] for the criteria resulting from Step 2. Performance was based on semi-structured interviews with health preference method experts and supplemented, where needed, by peer-reviewed literature [8, 13]. Each expert was asked whether a certain method could, as typically applied, meet each criterion. The expert replied with a “Yes”, “No”, “Maybe” or “Unsure” answer, and an explanation of their reasoning. Literature was used to complete any missing information, to turn the “Maybe” expert answers into definitive “Yes” or “No” by identifying the most common practice in the literature, to help make a definitive decision when experts could not reach a consensus or a majority (e.g. three expert answers of “Yes”, “No” and “Maybe”), or when expert opinion directly contradicted published literature.

### Step 4: Method Comparison

Each method was awarded assigned a performance score (*P*) by summing the weights times an indicator function for meeting the criteria. This is summarised in Eq. 1:

1 $$ P={\sum}_{i=1}^n{x}_i{y}_i $$

where *x*_*i*_ was the weight of the criterion (identified in Step 2), *y*_*i*_ was an indictor function that equals 1 if the method achieves criterion *i* or 0 if it does not (identified in Step 3), and *n* was total number of criteria, and *i* was the index of summation. By combining the weights determined from the AHP with the performance of each method, the 33 preference exploration and elicitation methods were compared. The higher the preference score, the more important criteria the method was able to meet.

The performance of the methods were compared within their designated taxonomy groups (Table 1) as defined by Soekhai et al. [12] to compare methods with similar approaches to data collection and analysis. Exploration and elicitation methods were compared separately because they are used under different circumstances to address different research questions and a significant number of the criteria (e.g. estimating trade-offs) were only suited for elicitation methods and would result in an undervaluation of all exploration methods. Additionally, methods were compared in their taxonomy groups in order to examine similar methods that may seek to answer similar research questions.

The method comparison was supplemented with information about the method’s publication frequency in peer-reviewed journals as applied to patient preferences, calculated from the systematic review of Soekhai et al. [12]. This was included after it was noted that several methods have not had any publications in the past few decades while others have had limited or no application to healthcare research. The methods’ publication frequency in peer-reviewed journals within the topic of patient preferences revealed that a total of 22 out of 33 of the methods had their most recent article concerning patient preferences published between 2012 and 2016. Several methods, such as measure of value, have not had any publications within healthcare contexts in the past few decades. Two methods, repertory grid method and starting known efficacy, were last published in 2005 and 1996, respectively. The remaining methods were not detected through systematic review. Other methods have had limited or no application to healthcare research, but experts have identified their potential (e.g. qualitative discriminant process [13]). Publication frequency was not used as a criterion used in Steps 1–3 because it would have been impossible for our participants and experts to know empirically how often a method is published without a systematic review.

## Results

### Step 1: Q-methodology

Out of 116 international stakeholders invited to participate, 54 participants (Additional file 1) completed at least one of the four online Q-methodology exercises and had their responses statistically analysed for similar viewpoints. Of the 35 initial criteria, 18 were identified as being most important for selecting a patient preference exploration or elicitation method each of the four hypothetical scenarios in the MPLC (see Table 3). These criteria obtained a positive average score (≥ 0.0) across all respondent groups with similar viewpoints, meaning the participants consistently ranked these criteria on the ‘important’ side of the grid. The results indicated that not all method criteria were important, or even relevant, for addressing stakeholder needs at different stages of the MPLC. For example, the cost of a preference study was thought to be an important criterion in both early development scenarios of the Q-methodology. However, it ranked low during the post-marketing scenario, and even lower in the phase III scenario, with six participants giving it the lowest possible ranking position.

### Step 2: Analytical Hierarchy Process

Out of 210 international stakeholders invited to participate, 85 participants (Additional file 1) completed at least one of their two designated exercises. Thirty-seven participants also completed the Q-methodology in step 1, although this had different objectives and was completed 4 months earlier. Our results showed that the relative importance of each criterion, as expressed by derived weights, is different for the particular stage in the MPLC where it will be performed, and the anticipated needs of the decision-maker that are specific to the scenario (Table 4). Establishing validity and reliability, as well as ensuring a low patient burden, received the highest weights in every stage of the MPLC. Cost, study duration, and sample size were very important in early development, particularly when the mechanism of action was known (Scenario A), although cost was less important than the other two when the mechanism was not known (Scenario B). Additionally, early stages demanded the exploration of reasons behind a preference in qualitative detail. Establishing and quantifying heterogeneity became more important in later stages, particularly during phase III. Also in this stage, the ability to estimate trade-offs was particularly important, more so than any other stage. Post-marketing had similar priorities to phase III, but the criteria of study duration and sample size were included in this Scenario D survey, and received a significant share of the total weights.

**Table 4:** Criteria weights (%) for each Scenario (A-D) determined from the AHP

**Criteria**	A: Early development	B: Early development	C: Late phase III	D: Post-marketing
Cost	12.38	10.36		
Sample Size	11.76	12.91		14.01
Study duration (time needed)	12.10	13.18		14.36
Low frequency of sessions	5.45	4.21	–	–
A low cognitive load on patients	8.21	4.35	–	–
Quick sessions with participants	–	2.04	–	–
Complexity of instructions to participants	–	3.78		–
Group dynamic with participants	–	–	1.95	–
No interaction between participants (Solitarily exercise)	–	–	3.80	–
Ease to which new attributes can be added without making prior results invalid	2.91	2.75	2.92	–
Estimating weights for attributes	4.60	3.59	6.45	4.04
Estimating trade-offs between attributes	5.48	6.18	9.31	5.98
8 or more attributes can be explored	–	–	–	1.89
Degree to which internal validation methods can be incorporated	7.16	8.87	12.89	7.57
Degree to which external validity is established	10.15	8.00	11.72	11.62
Exploring the reasons behind a preference in qualitative detail	8.00	9.01	6.09	4.91
Public acknowledgement by your organisation as an acceptable method to study preferences	–	–	6.15	4.27
Quantifying heterogeneity in preferences	6.94	6.62	13.2	9.02
Calculating of risk attitudes (like risk tolerance vs. risk aversion) due to uncertainty in the value of an attribute	4.87	4.18	8.36	6.85

### Step 3: Method Performance

The performance of each method under the criteria was determined by consulting international preference method experts (*n* = 17) (Table 5). Six methods (complaints procedures, concept mapping, measure of value, starting known efficacy, outcome prioritization tool, and qualitative discriminant process) were informed exclusively by literature because no method expert could be contacted at the time of analysis. A sensitivity analysis was conducted if there was ultimately no clear consensus. There was a lack of consensus among the experts for the performance of best-worst scaling type 1, the performance of (probabilistic) threshold technique, and whether most methods could be performed in a group setting, so literature was consulted as a tie-breaker, and to determine how the methods performed typically, and not what they could theoretically achieve in a hypothetical sense.

**Table 5:**
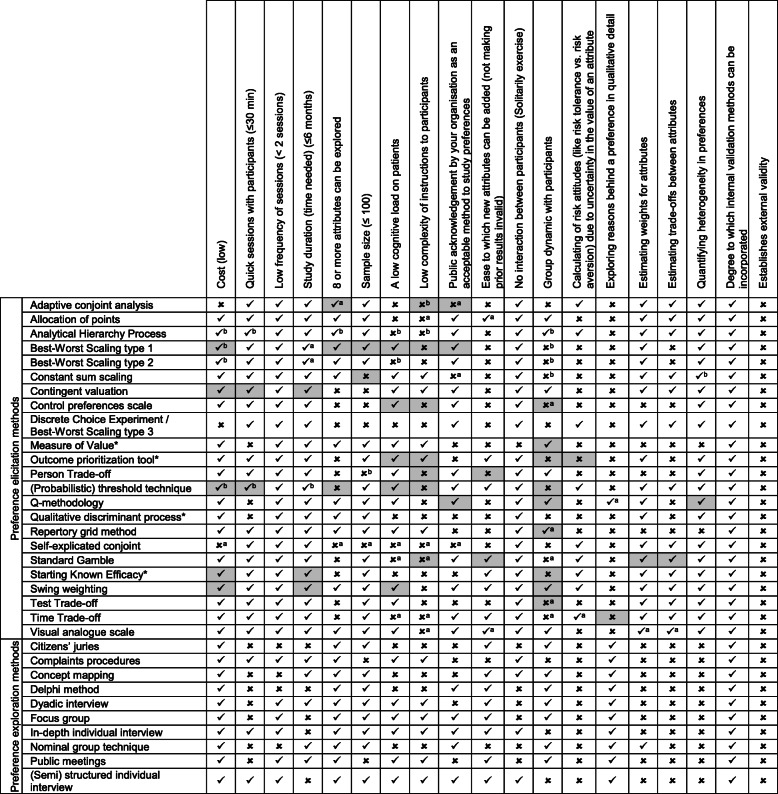
Method performance

✓ = meets criterion; ✘ = does not meet criterion; **Grey** = indicates a lack of unanimous consensus among the experts;

*Informed exclusively by literature, and not expert interviews;

^a^Literature conflicted with experts

^b^No clear majority. Literature broke the tie

### Step 4: method comparison

**Fig. 2:**
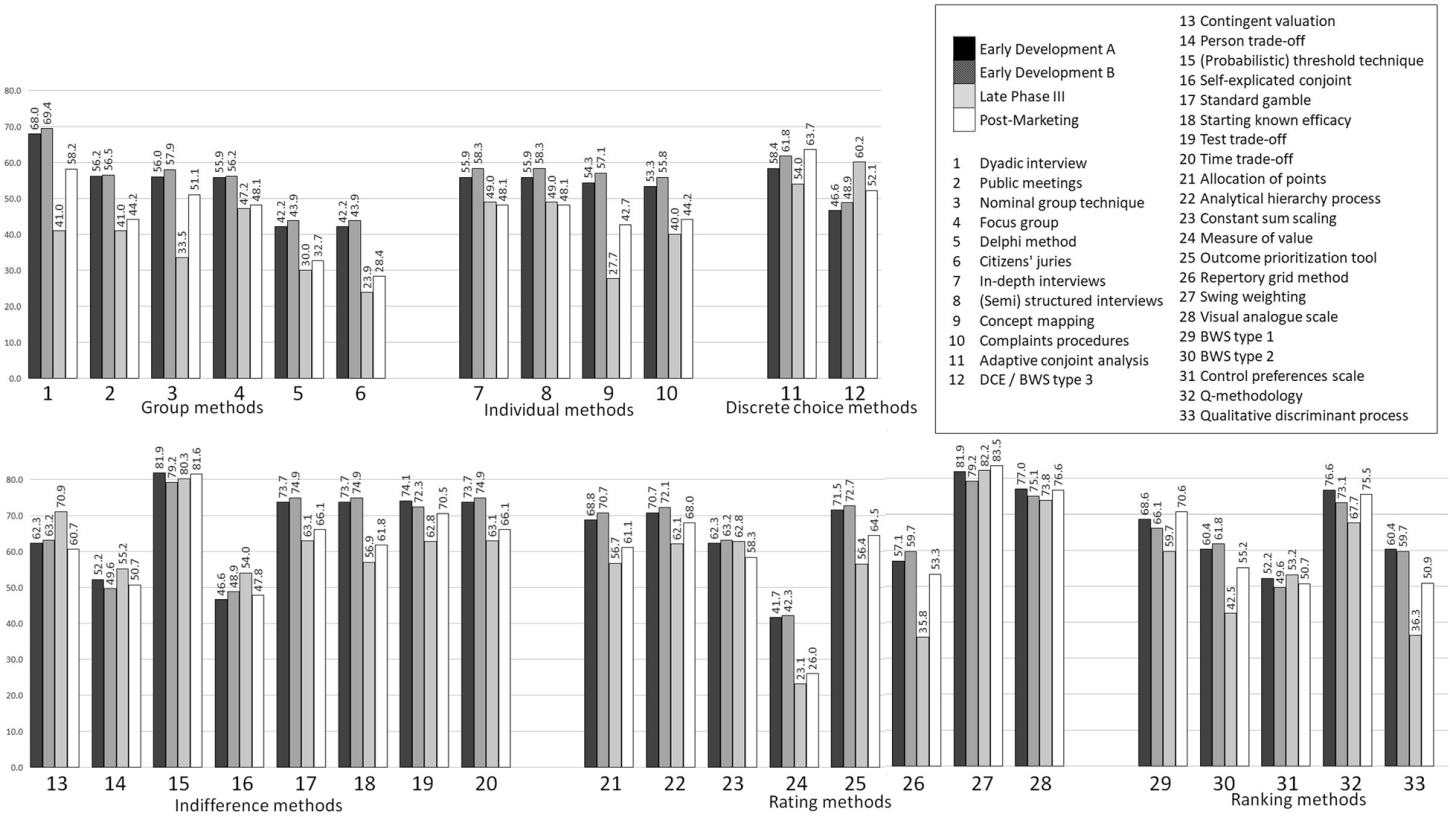
Method comparison

The performance of each method was closely examined by comparing their scores for the different stages of the MPLC, compared to other methods’ scores within the same taxonomy group (Fig. 2). Publication frequency was also considered when assessing the method’s performance, after it was noted that several methods have not had any publications over the past 20 years, or have had limited application in healthcare research. Therefore, this additional information helped contextualise the performance of the methods in a real-world context.

**Fig. 3:**
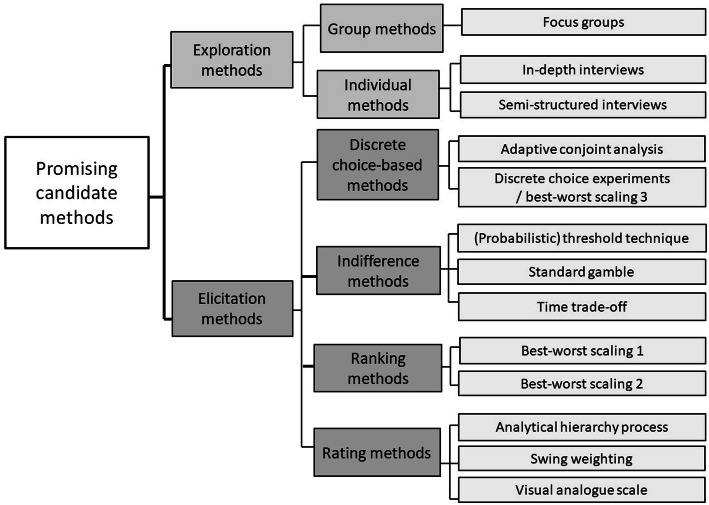
Thirteen most promising methods to explore or elicit patient preferences

A total of 13 elicitation and exploration methods were identified as *promising*, meaning they are most likely to meet most decision-makers’ needs during all stages of the MPLC. These methods obtained the highest overall scores for all stages of the MPLC, relative to other methods within the same taxonomy group. For the exploration methods, these include focus groups, in-depth interviews, and semi-structured interviews (Fig. 3). For the elicitation methods, these include discrete choice experiments / best-worst scaling type 3 (DCE/BWS3), adaptive conjoint analysis, (probabilistic) threshold technique, standard gamble, time trade-off, best-worst scaling type 1 (BWS1), best-worst scaling type 2 (BWS2), swing weighting, visual analogue scale, and analytical hierarchy process (AHP) (Fig. 3). Rather than identifying only one overall highest scorer, we identified several instances of more than one promising method within the same taxonomy group.

Additionally, eight other methods were identified that may have *potential* but only for some of the MPLC stages, or might have some publication frequency issues of which decision-makers must be aware before selecting these methods. This does not necessarily mean that the method would never be successful, but decision-makers need to balance the identified benefits and risks. For exploration methods, these are nominal group technique, public meetings, and dyadic interviews. For elicitation methods, these include test trade-off, starting known efficacy, Q-methodology, outcome prioritization tool, and constant sum scaling.

### Group exploration methods

Group exploration methods collect data from more than one participant in a single session [24–26]. Most of these methods performed better during early development than in later stages. Dyadic interviews, focus groups, nominal group technique, and public meetings were the highest performing group exploration methods likely to meet most decision-makers’ needs during all stages of the MPLC. However, dyadic interviews were not detected through systematic review [12]. Focus groups, on the other hand, performed strongly across all stages and were the most well-published group method [12]. Nominal group technique appears promising only for early development and post-marketing. Public meetings appear promising for early development and late phase III. Despite a low publication frequency, this method is frequently employed by the FDA during early development as a method of patient engagement [27]. However, this method has been criticised for a lack of robustness [28]. All group methods did not perform very well in late phase III; probably because this phase had criteria reflecting decision-makers’ needs for quantitative PP. Delphi method and citizens’ juries did not perform as well across all phases, because they had relatively higher cognitive burdens, more sessions for patients, and a longer study duration. However, the Delphi method has a relatively strong publication frequency [12]. In all, focus groups appeared to be the most *promising* group exploration method likely to meet most decision-makers’ needs during all stages of the MPLC. Nominal group technique, public meetings, and dyadic interviews are also *potential* group exploration methods because they can achieve some decision-makers’ needs during particular stages of the MPLC.

### Individual exploration methods

Individual exploration methods collect data from one participant in a single session [29, 30]. In-depth interviews and semi-structured interviews were the highest performing methods in this category. Concept mapping and complaints procedures did not perform as well, and their performance was derived exclusively from literature. Therefore, in-depth interviews and semi-structured interviews appeared to be the most *promising* group exploration methods. No *potential* methods were identified in this group.

### Discrete choice based elicitation methods

Discrete choice based elicitation methods examine the relative importance of trade-offs between attributes and their alternatives through a series of hypothetical choices [31, 32]. Although there were only two methods in this category, both discrete choice experiments / best-worst scaling type 3 (DCE/BWS3) and adaptive conjoint analysis both performed highly, both inside their group and relatively across all methods. Adaptive conjoint analysis has some well-published theoretical concerns, both structural and statistical [33, 34] that decision makers should be aware of before selecting it, as well as a lower publication frequency compared to DCE/BWS3. Nonetheless, DCE/BWS3 and adaptive conjoint analysis appear to be the most *promising* discrete choice based elicitation methods.

### Indifference elicitation methods

Indifference elicitation methods use techniques that examines a participant’s preferences for one attribute or alternative over another, until the participant is indifferent or has no preference [35, 36]. (Probabilistic) threshold technique, standard gamble, and time trade-off performed the best out of this group. Test trade-off could be promising in early development when the mechanism of action is known, and post-marketing, but this method could not be detected through systematic review [12]. Starting known efficacy also appeared promising for early development stages, although this result was based on literature, and not expert interviews. Contingent valuation does not perform as well compared to the other methods, despite a positive publication frequency. It appears to be a promising method during phase III, possibly due to its ability to satisfy vital criteria for this stage: estimating weights for attributes and trade-offs between attributes whilst still maintaining a relatively low cognitive burden. Person trade-off did not perform well for any stage, possibly because of its sample size requirements and limitations for what its outcomes can estimate. Both these methods also had the least amount of expert consensus (see Table 5). In summary, (probabilistic) threshold technique, standard gamble, and time trade-off appeared to be the most *promising* indifference elicitation methods likely to meet most decision-makers’ needs during all stages of the MPLC. Additionally, test trade-off and starting known efficacy could also be *potential* methods during particular stages of the MPLC.

### Rating elicitation methods

Rating elicitation methods use comparative rating approaches [37, 38]. Swing weighting, visual analogue scale (VAS), and analytical hierarchy process (AHP) performed the best out of this group. AHP was evaluated as a (rating) elicitation method in this study (see Fig. 1), in addition to being used as part of the methodology itself (Step 2). Interestingly, swing weighting performed the best out of all elicitation methods, across all groups. Constant sum scaling performed well for late phase III, but it could not be detected through systematic review [12]. Outcome prioritization tool appears to be promising for early development and post-marketing, but its performance was informed exclusively by literature and not expert interviews. Allocation of points, constant sum scaling, and repertory grid method performed worse than other methods in the group. This is because they did not satisfy several important criteria (such as calculating risk attitudes) and their publication frequency was lower than the others. Measure of value could not be detected through systematic review [12]. In summary, swing weighting, VAS and AHP appear to be the most *promising* rating elicitation methods likely to meet most decision-makers’ needs during all stages of the MPLC. Additionally, outcome prioritization tool could also be a *potential* method, as could constant sum scaling during phase III.

### Ranking elicitation methods

Ranking elicitation methods use ranking exercises [39, 40]. Best-worst scaling type 1 (BWS1), best-worst scaling type 2 (BWS2), and Q-methodology performed the best out of this group. However, Q-methodology was not detected through systematic review [12], probably due to it being a relatively new method in healthcare research. Qualitative discriminant process could be promising in early development when the mechanism of action is known, although it was also not detected through systematic review, and its performance was informed exclusively by literature and not expert interviews. Additionally, other reviews have noted its lack of application to healthcare research [13]. Self-explicated conjoint analysis could not be detected through systematic review and did not perform as well as the other ranking methods [12]. Control preferences scale could be promising for late phase III, despite a moderately low publication score, although it performed consistently low for the other stages. This is likely because the method has had few applications in healthcare, let alone patient preferences [13]. In summary, BWS1 and BWS2 appear to be the *promising* ranking elicitation methods most likely to meet most decision-makers’ needs during all stages of the MPLC. Additionally, Q-methodology could also be a *potential* method if decision-makers are willing to compromise on its rate of publication in recent years.

## Discussion

Through a four-step approach, this study identified 13 exploration and elicitation methods most suitable for patient preference studies at different stages of the MPLC. By applying the numerical weights calculated for each important criterion in this study, decision-makers can potentially be aided when selecting a method. A total of 13 elicitation and exploration methods were identified as *promising*, meaning they are most likely to meet most decision-makers’ needs during all stages of the MPLC (Fig. 2). For the exploration methods, these include focus groups, in-depth interviews, and semi-structured interviews (Fig. 3). For the elicitation methods, these include DCE/BWS3, adaptive conjoint analysis, (probabilistic) threshold technique, standard gamble, time trade-off, BWS1, BWS2, swing weighting, visual analogue scale, and AHP (Fig. 3).

### Strengths and limitations

There is currently no comprehensive overview of how to directly compare patient preference methods or how to determine which are more suitable for decision-makers’ needs. Decision-makers would benefit from having such information to improve the systematic inclusion of patient preferences throughout the MPLC. The key strength of this study is its empirical approach with the direct comparison of many diverse methods and the significant contribution by international health preference experts. Other appraisals of patient preference methods have been conducted [13, 41], although we have attempted to be more systematic, quantitative, and up-to-date. Another strength is its transparency, incorporating several tools for examining method performance, including the criteria and weights established by a Q-methodology, and an AHP.

There are limitations to our sample. Although our response rate was lower than expected, both the Q-methodology and the AHP do not require large sample sizes. Therefore, we were still able to conduct a meaningful statistical analysis with *n* = 54 and *n* = 85 participants, respectively. In terms of representativeness, these results may not be generalizable to the larger sample of preference methodologists. There were significantly more industry members and academics in both the Q-methodology and AHP, with an under-representation of HTA/payer representatives and regulators. These latter two groups could have made a significant contribution by sharing insights into the requirements of patient preference study design during health technology assessments or during market authorisation. Most patient organization representatives and physicians did not fulfil the requirements of having patient preference method experience or have sufficient understanding of MPLC decision-contexts and were therefore not included in the analysed data. The methodological and technical focus of this appraisal meant that actual experience with these methods was crucial.. Despite limitations of our sample, the international diversity of all cohorts was significant and a relatively high consensus was achieved among them in terms of the identification of more promising preference methods in each taxonomy category.

All four of the MPLC scenarios created for both the Q-methodology and AHP relate strongly to industry decision points. They were written in a way to be accessible to persons with little pharmaceutical development experience, and they contained a variety of possible situations (e.g. certainty or uncertainty concerning the product, internal or external submissions, and early or late stages in development) applicable to other MPLC decision points not tested in this task, such as specific HTA/payer or regulatory decision points. The scenarios were not meant to stand in as proxy for decision-maker objectives and research questions; there is a range of potential research questions that decision-makers could seek to answer at each stage. However, they do offer insight into some real-world decision-points. For example, we included a scenario in which the goal is to provide patient preference data to support benefit-risk assessment when submitting dossiers to regulators or HTA bodies. We examined which needs were most important to decision-makers during this situation (reflected in our criteria) and identified the methods most likely to meet these needs during this specific situation, and help create a successful dossier submission.

The incorporation of method taxonomies helps decision-makers identify the most suitable method that would best aim to answer their research question. However, this study does not intend to assist decision-makers with determining the taxonomy group that is most appropriate to answer a specific research question; this is out of scope for this study.

It is not as accurate to compare the weights of the same attribute in different scenarios directly because there were different numbers of attributes in each scenario. As the Q-methodology indicated, not all method criteria were important, or even relevant, at every stage of the MPLC. Evaluating every criterion from the original list of 35 through an AHP would have been a highly burdensome pairwise comparison exercise. It was not possible to re-combine the criteria identified through the Q-methodology into one large AHP survey, because it would have defeated the purpose of identifying MPLC-specific criteria. For example, the cost of a preference study was thought to be an important criterion in both early development scenarios but very unimportant in later stages. Therefore, it would be illogical, and unnecessary participant burden, to use this criterion in an attribute for Scenarios C and D during the AHP.

For the AHP, a relatively common practice is to check the consistency of each judgement made by participants to ensure a reasonable level of consistency in terms of proportionality and transitivity [42]. However, given the large size of data to be analysed, and the number of judgements made across all the scenarios, it was not feasible to check the consistency of every judgement. This could be a beneficial addition to future sensitivity analysis. Several academic studies have indicated that weight derivation from pairwise comparisons is much more accurate than direct weighting [43], although three participants commented that the 7-point scale of the AHP was unnecessarily large. Additionally, the quantification of a “low” survey cost, a “small” sample size, or a “short” study duration are highly subjective, and we cannot control participant perceptions of these quantities. However, we instructed participants to not focus on a specific amount, but rather ask how important this concept (e.g. cost) is, in general, at this stage.

The application of the AHP weights to the method performance was a complex process, and the construction of the performance grid (Table 5) was an ambitious task. A total of 17 international experts were contacted over a period of 5 months. It was originally planned to have at least 3 different experts contribute to each method, cross-verifying the data. However, this was not always feasible, meaning additional literature was consulted to fill the gaps. Disagreement from experts in this innovative field is not unexpected, and this study provided a platform for an engaged discussion. Some of our consulted experts argued that reducing a method’s capabilities to a binary “yes” or “no” answer eliminates shades of grey. In many cases, a method is *capable* of being conducted a particular way under certain circumstances (e.g. using a very small sample size, although this may compromise reliability and reproducibility). In these cases, we decided to examine what was *typical* or *common practice* for the method, instead of what the method could achieve in the hypothetical sense. This is one of the key motivations for augmenting these scores with the literature results, as it demonstrated how this method has been observed to behave in the field of preference elicitation or exploration.

Although tested in a relatively small setting, this novel approach warrants further development in the future. Even if a method obtains a low performance score using our approach, it does not necessarily mean that it could never meet a decision maker’s needs in the right circumstances. It is possible to repeat variations of these four-steps with different samples of participants (e.g. industry members exclusively) and different MPLC scenarios or in order to determine stakeholder-specific criteria, creating a tailored short list of suitable methods for their unique situation (e.g. informing cost-effectiveness ratios). This study ultimately reflects one example of how this approach be accomplished. By utilising the steps included in this study, either individually or as a whole, decision-makers have a tool for selecting an exploration or elicitation method most suited to their needs. Future research building upon this study could help develop a decision-tree for different stakeholders to give guidance of which method is most useful for a certain research question. Other research should investigate whether patient preference data should be directly incorporated within an economic evaluation or as additional information alongside an economic evaluation.

## Conclusion

This study aimed to develop criteria to characterise and appraise preference exploration and elicitation methods, and create a comprehensive overview based on empirical evidence of how these methods compare to one another within the MPLC. A total of 13 elicitation and exploration methods were identified as suitable and most likely to meet most decision-makers’ needs during all stages of the MPLC. Additionally, we identified eight methods that could have potential for some of these stages, although we have identified potential issues of which decision-makers should be aware before selecting these methods. Our rigorous, quantitative review of preference methods provides guidance for decision-makers to consider when selecting a method for a patient preference study.

## Supplementary information

**Additional file 1.** Appendix I: Participant demographics and stakeholder affiliation.

## Data Availability

The datasets during and/or analysed during the current study available from the corresponding author on reasonable request.

## Abbreviations

AHP: Analytical hierarchy process
BWS1: Best-worst scaling type 1
BWS2: Best-worst scaling type 2
BWS3: Best-worst scaling type 3
CDRH: Center for Devices and Radiological Health
DCE: Discrete choice experiments
EMA: European Medicines Agency
FDA: Food and Drug Administration
HPR: Health preference research
HTA: Health technology assessment
MPLC: Medical product life cycle
NICE: National Institute for Health and Care Excellence
PP: Patient preference
PREFER: Patient Preferences in Benefit-Risk Assessments during the Drug Life Cycle
US: United States
VAS: Visual analogue scale
