# Supplementary material for: Appraising patient preference methods for decision-making in the medical product lifecycle: an empirical comparison
Source: BMC Med Inform Decis Mak. 2020 Jun 19;20:114. doi: 10.1186/s12911-020-01142-w (PMC7304129; doi:10.1186/s12911-020-01142-w)
Supplement: Supplementary file 1 — Additional file 1. Appendix I: Participant demographics and stakeholder affiliation. [file 12911_2020_1142_MOESM1_ESM.docx]

Supplemental File: Appendices for “Appraising patient preference methods for decision-making in the medical product lifecycle: An empirical comparison”

Chiara Whichello (whichello@eshpm.eur.nl)^1^, Bennett Levitan B^2^, Juhaeri Juhaeri^3^, Vaishali Patadia^3^, Rachael DiSantostefano^2^, Cathy Anne Pinto^4^, Esther W. de Bekker-Grob^1^

^1^ Erasmus School of Health Policy & Management and Erasmus Choice Modelling Centre, Erasmus University Rotterdam, Rotterdam, the Netherlands

^2^Janssen R&D, LLC, United States of America
^3^ Sanofi, United States of America

^4^ Merck & Co, Inc, United States of America

Appendix I: Participant demographics and stakeholder affiliation

**Participant demographics and stakeholder affiliation (Q-methodology and AHP)**

|  |  | Q-methodology | | | | AHP | | | |
| --- | --- | --- | --- | --- | --- | --- | --- | --- | --- |
|  |  | Early Development A  n=36 | Early Development B  n=28 | Late Phase III n=31 | Post-Marketing n=28 | Early Development A  n=39 | Early Development B  n=46 | Late Phase III n=37 | Post-Marketing n=50 |
| Stakeholder | AC | 8 | 4 | 7 | 3 | 16 | 28 | 20 | 17 |
|  | CO | 2 | 1 | 1 | 1 | 3 | 1 | 1 | 5 |
|  | HT | 1 | 1 | 0 | 1 | 1 | 0 | 0 | 1 |
|  | IN | 22 | 19 | 20 | 19 | 16 | 14 | 11 | 22 |
|  | PH | 1 | 2 | 1 | 2 | 1 | 1 | 2 | 0 |
|  | PO | 2 | 0 | 2 | 0 | 1 | 0 | 1 | 0 |
|  | RE | 0 | 0 | 0 | 0 | 0 | 1 | 1 | 0 |
|  | O* | 0 | 1 | 0 | 2 | 1 | 2 | 1 | 1 |
| Country | BE | 5 | 0 | 3 | 0 | 4 | 0 | 0 | 4 |
|  | CH | 2 | 4 | 1 | 4 | 1 | 2 | 1 | 2 |
|  | DE | 5 | 5 | 4 | 5 | 5 | 7 | 4 | 8 |
|  | IT | 2 | 1 | 2 | 0 | 2 | 0 | 2 | 0 |
|  | NL | 3 | 1 | 3 | 1 | 7 | 7 | 9 | 5 |
|  | SE | 2 | 1 | 2 | 2 | 0 | 3 | 1 | 1 |
|  | UK | 5 | 5 | 3 | 5 | 4 | 9 | 7 | 6 |
|  | US | 11 | 11 | 9 | 10 | 15 | 14 | 12 | 15 |
|  | FR | 0 | 0 | 0 | 0 | 0 | 1 | 0 | 1 |
|  | O** | 1 | 0 | 4 | 1 | 1 | 5 | 1 | 4 |

AC = academia; CO = consultant; HT = health technology assessment body or payer; IN = industry; PH = physicians; PO = patient organisation members; RE = regulators; O = Other; BE = Belgium; CH = Switzerland; DE = Germany; IT = Italy; NL = Netherlands; SE = Sweden; UK = United Kingdom; US = United States; FR = France; AHP = Analytical hierarchy process

*Other includes: Independent research organisation, International organisation, and University hospital

**Other includes: Australia, Canada, Norway, Poland, and Spain
